# Intravoxel Incoherent Motion Diffusion-weighted MR Imaging for Monitoring the Therapeutic Efficacy of Interventional Photothermal Therapy with Nanoparticles in Rabbit VX2 Tumors

**DOI:** 10.2174/0115734056401967251203063844

**Published:** 2026-01-16

**Authors:** Jia Cao, Jun Zhou, Gonghao Ling, Qingyun Long

**Affiliations:** 1 Department of Radiology, The Central Hospital of Wuhan, Tongji Medical College, Huazhong University of Science and Technology, Wuhan 430014, China; 2 Department of Radiology, Zhongnan Hospital of Wuhan University, Wuhan, China

**Keywords:** Diffusion-weighted imaging, Intravoxel incoherent motion, Nanoparticles, VX2 tumor, Transcatheter intra-arterial infusion, Photothermal therapy

## Abstract

**Introduction:**

In this study, we evaluated the efficacy of transcatheter intra-arterial infusion of lecithin-modified Bi-Ln nanoparticles (Bi-Ln NPs) combined with interventional photothermal therapy (IPTT) using a rabbit VX2 tumor model, employing intravoxel incoherent motion diffusion-weighted imaging (IVIM-DWI) for assessment.

**Methods:**

Thirty-two rabbit liver VX2 tumor models were established, and transcatheter intra-arterial infusion of Bi-Ln NPs was performed using superselective intubation under digital subtraction angiography (DSA) guidance. IPTT was then carried out by inserting a near-infrared (NIR) optical fiber into the rabbit VX2 tumors under real-time ultrasound guidance. Magnetic resonance imaging (MRI) was performed one day before treatment and seven days after treatment to evaluate therapeutic efficacy, using T1-weighted imaging (T1WI), T2-weighted imaging (T2WI), gadolinium-enhanced T1WI, and IVIM-DWI. After treatment, gross and histopathological examinations were conducted to categorize liver tumors into viable tumor, inflammatory reaction, and necrotic regions. IVIM-derived parameters were calculated and compared across these regions. Additionally, immunohistochemical analysis was performed to further assess treatment efficacy.

**Results:**

The tumor-bearing rabbits exhibited significant therapeutic effects, as shown by comparative analysis of MRI images and parameters before and after treatment. Both the mean apparent diffusion coefficient (ADC) and diffusion coefficient (D) increased significantly after treatment (*P* = 0.008 and *P* = 0.034, respectively). Pathological analysis also revealed an elevated apoptosis rate of tumor cells, with a mean of 43.26 ± 12.26%. Across the different lesion regions, the ADC and D values were significantly lower in the viable tumor region than in the inflammatory reaction region (both *P* < 0.001). However, the D* values in the viable tumor region did not differ significantly from those in the inflammatory reaction region. Additionally, the ADC, D, and f values were significantly reduced in the necrotic region compared with the inflammatory reaction region (*P* = 0.003, <0.001, and <0.001, respectively). In the receiver operating characteristic (ROC) analysis, the diffusion coefficient (D) demonstrated the highest area under the curve for distinguishing between the inflammatory reaction and viable tumor regions.

**Discussion:**

IVIM-DWI demonstrates strong potential for detecting early tumor responses to therapeutic interventions and for differentiating tissue types following treatment. The parameters derived from this technique may provide preliminary insight into therapy-induced physiological changes.

**Conclusion:**

The combination of transcatheter intra-arterial infusion and IPTT represents a promising strategy for effective tumor eradication, thereby improving therapeutic outcomes. IVIM-DWI offers a quantitative tool for monitoring early treatment responses in hepatic tumors and distinguishing between different tissue types after therapy.

## 1 INTRODUCTION

Hepatocellular carcinoma (HCC) ranks as the third leading cause of cancer-related mortality, with liver metastases frequently occurring in solid malignancies, often signifying a poorer prognosis [1, 2]. The management of patients with primary or metastatic liver tumors remains complex, as local tumor recurrence and distant metastasis continue to pose significant challenges [3]. Currently, radiofrequency ablation (RFA) is associated with a high rate of local tumor recurrence, and systemic chemotherapy is constrained by substantial toxicity and limited efficacy. Liver transplantation, while effective, is hindered by high costs and a shortage of donors, resulting in less than 20% of eligible patients undergoing the procedure [4, 5]. Transhepatic arterial chemoembolization (TACE) and radioembolization are minimally invasive interventional radiology techniques that offer promising and innovative routes for the administration of theranostic agents to tumors. To address the issues of low tumor targeting and suboptimal therapeutic efficacy in the treatment of liver tumors, we propose the integration of interventional photothermal therapy (IPTT) with transcatheter intra-arterial infusion to deliver therapeutic agents directly to tumor sites, thereby enhancing cancer cell eradication. PTT utilizes light-absorbing agents to induce heat and achieve thermal ablation of tumors while sparing healthy tissues, making it an effective, noninvasive, and highly targeted cancer treatment [6-10]. PTT presents several advantages, including the use of external laser irradiation with adjustable dosage, which allows for precise targeting of tumors and minimizes damage to surrounding healthy tissues [11-16]. Nevertheless, a significant limitation of PTT is the restricted depth of light penetration, which may result in incomplete ablation of tumors located beyond the irradiation range. Consequently, in order to enhance the therapeutic efficacy of PTT for deep-seated tumors, we have opted to utilize real-time ultrasound-guided IPTT. Then, the therapeutic outcome was evaluated and analyzed using current imaging techniques in a timely manner.

While changes in tumor size have traditionally been employed to assess treatment response, such measurements may not reflect immediate changes, thereby limiting their ability to accurately and promptly evaluate the effects of anticancer therapies. Consequently, functional imaging techniques may offer more precise alternatives for assessing treatment efficacy. At present, DWI and the derived ADC provide valuable insights into tumor cellularity and necrosis [17, 18]. However, to date, no studies have investigated the use of IVIM-DWI for evaluating the efficacy of transcatheter intra-arterial infusion with IPTT therapy. Therefore, this study seeks to quantitatively assess the therapeutic effects of IVIM-DWI on transcatheter intra-arterial infusion and IPTT in rabbit VX2 liver tumors.

## 2 MATERIALS AND METHODS

### 2.1 Agents

Bismuth nanoparticles (Bi NPs) were synthesized following the methodology of Wu *et al*. [19], with minormodifications. The Bi-Ln NPs were prepared *via* emulsification techniques. The synthesized Bi-Ln NPs were subsequently dispersed in water and stored at 4°C for further analysis. In the experimental procedure, Bi-Ln NPs (4 mg/ml, 3 ml) and phosphate-buffered saline (PBS, 3 ml) were effectively administered into the arterial supply of a rabbit tumor using a microcatheter.

### 2.2 Animal Tumor Model

VX2 tumor tissues were harvested from VX2 tumor-bearing rabbits supplied by the Laboratory Animal Research Center at Tongji Medical College, Huazhong University of Science and Technology. A cohort of thirty-two Japanese big-ear rabbits (equal numbers of males and females) was obtained from Wuhan Wanqianjiaxing Biotechnology Co., Ltd. (Wuhan, China). The rabbits were 6 months old and weighed 2.5–3.4 kg.

Anesthesia was administered *via* slow intravenous injection of 3% pentobarbital sodium (1 ml/kg) through the ear vein. The upper abdominal region of each rabbit was shaved and prepared for aseptic surgical procedures. A mini-laparotomy was performed, and VX2 tumors were inoculated into the left hepatic lobe using 1–2 mm fragments of freshly excised VX2 tumor tissue. The incision was closed in layers to ensure proper healing and recovery.

Tumor growth in the liver was allowed to progress for approximately two weeks, after which MRI scans were conducted to evaluate VX2 tumor development. The implanted tumor masses reached an average size of 1–2 cm.

The selection criteria for an appropriate tumor model for treatment were as follows:

- a single tumor nodule located within the liver parenchyma;
- a tumor of suitable size (not exceeding 3 cm);
- minimal necrotic tissue, accounting for only a small portion of the total tumor area;
- no evidence of tumor penetration through the hepatic capsule;
- absence of tumor metastasis in the peritoneum or adjacent abdominal organs.

Exclusion criteria: The tumor protrudes beyond the surface of the liver, with the maximum diameter of the tumor exceeding 3 cm; the necrotic area accounts for more than half of the total area of the tumor, and metastasis is found in adjacent organs.

According to these criteria, 30 tumor-bearing rabbits were included in the subsequent experiments. This study was approved by the Animal Ethics Committee of Wuhan University.

### 2.3 Interventional Procedures

For the intracatheter infusion, a DSA system (Allura Xper FD20, Philips Healthcare, Netherlands) was used. Anesthesia was induced with 3% sodium pentobarbital at a dosage of 1 ml/kg. After standard disinfection, a small incision was made to expose the rabbit’s right femoral artery. Using a 4-F vascular sheath, the artery was punctured, and a 4-F catheter was advanced to the common abdominal artery under DSA guidance. A contrast agent (Iodixanol Injection) was then injected to visualize the hepatic arteries. Subsequently, a 2.7-F microcatheter (Terumo) was inserted into the left hepatic artery. Under fluoroscopic monitoring, the tumor and its supplying arteries were accurately identified.

Bi-Ln NPs (4 mg/ml, 3 ml) and PBS (3 ml) were smoothly administered through the microcatheter positioned in the tumor-feeding artery. After the infusion, the femoral artery was ligated, and the incision was sutured following catheter removal.

IPTT was performed after a 5-minute interval under real-time ultrasound guidance using a NIR optical fiber (China). An 18-gauge PTC needle was used to access the liver tumor, which was then exposed to 808-nm laser irradiation at a power density of 1.0 W/cm^2^ for 3 minutes. To maintain consistent positioning throughout the procedure, the rabbits were secured to a rigid board.

A schematic diagram illustrating the effective eradication of hepatic tumors using the combined transcatheter intra-arterial infusion and IPTT approach is shown in Fig. (**1**).

### 2.4 MRI Examination and Image Analysis

All images were acquired using a 3.0 Tesla MR scanner (Prisma, Siemens, Erlangen, Germany) equipped with a human knee coil. Anesthesia was administered *via* slow intravenous injection of 3% pentobarbital sodium (1 ml/kg) through the auricular vein. The rabbits were positioned prone, and an abdominal band was used to apply gentle pressure and stabilize the abdomen.

Liver scans were performed using the BLADE sequence to obtain transverse BLADE turbo spin echo T2-weighted images, minimizing motion artifacts and ensuring high image clarity. The MRI protocol included T1-weighted imaging (T1WI), T2-weighted imaging (T2WI), gadolinium-enhanced T1WI, and DWI, with the following parameters: repetition time (TR) of 2,300 ms, echo time (TE) of 104 ms, and a section thickness of 3 mm.

IVIM-DWI images were acquired during free breathing, using b values of 0, 50, 100, 150, 200, 250, 300, 500, 800, 1000, and 1500 s/mm^2^. Scans were conducted both before treatment and one week after treatment. Gadolinium-enhanced T1WI was obtained through rapid administration of a gadolinium chelate *via* the ear vein.

Image analysis was performed using conventional MRI sequences in combination with IVIM-DWI.

The ADC was determined using a monoexponential fitting model incorporating all 11 b-values. The relationship between signal variation and the b-factor is represented by the equation: S(b)/S_ 0_ = e^-bADC^, where S_b_ denotes the signal intensity at a specific b-value, and S_0 _ represents the signal intensity in the absence of a diffusion gradient (b=0). In accordance with IVIM theory, the parameters diffusion coefficient (D), perfusion-related diffusion coefficient (D*), and perfusion fraction (f) were estimated using a biexponential fitting model, as described by the equation: S(b)/S_ 0_=(1-f).e^-bD^+f.e^-bD*^.

In the transverse T1WI, T2WI, and contrast-enhanced T1-weighted images, signal intensities were visually assessed in comparison to muscle tissue and categorized as hypointense, isointense, or hyperintense. Quantitative diffusion metrics were independently acquired for each lesion by calculating parameters from user-defined circular regions of interest (ROIs) ranging from approximately 10 to 30 mm^2^. This involved analyzing the characteristics of MRI and IVIM-derived parameters on the images and correlating histopathological results with imaging findings. The magnetic resonance images were evaluated collaboratively by two senior radiologists, with a conclusion reached by consensus.

### 2.5 Histological Evaluation

Following the completion of the MRI, all rabbits were euthanized, and liver specimens were collected. Each pathological specimen was sectioned in the same axial plane as the MR images and subsequently stained with hematoxylin-eosin (HE). Tumor tissue viability was evaluated using a TUNEL assay kit (Roche, Shanghai, China). The prepared slides were examined and imaged using an optical microscope (OLYMPUS IX51, Japan). TUNEL-positive cells, indicative of apoptosis, were quantified using Image J analysis software (version 1.8.0, USA). The apoptotic cell nuclei rate was calculated as the ratio of apoptotic positive cell nuclei to total cell nuclei within the field. Histopathological analysis was conducted by a senior pathologist who was blinded to the treatment conditions.

### 2.6 Statistical Analysis

All quantitative data are presented as mean ± standard deviation (SD). The normality of the DWI-IVIM parameters was assessed using the Shapiro-Wilk test, with a *P*-value greater than 0.05 indicating a normal distribution. Homogeneity of variance was evaluated using Levene's test. The Kruskal-Wallis test was employed to compare the ADC, D, D*, and f values across viable, necrotic, and inflammatory regions. Subsequently, ROC analysis was conducted to assess the diagnostic performance of ADC, D, and f in differentiating between viable tumor regions and inflammatory reaction regions. The cutoff values for ADC, D, and f were determined based on the largest Youden index derived from the ROC curves. Sensitivity and specificity were calculated with exact 95% confidence intervals. A *P*-value less than 0.05 was considered indicative of a statistically significant difference. All comparative analyses were performed using SPSS version 22.0 (SPSS Inc., Chicago, IL, USA).

## 3 RESULTS

### 3.1 MRI Examination

Out of the 32 rabbit VX2 tumor models, 30 were successfully established, while two rabbits died due to anesthetic overdose. Consequently, 30 liver lesions were included in the final study cohort. Before treatment, all VX2 tumors displayed homogeneous isointensity on T1WI, relative hyperintensity on T2WI, hyperintensity on DWI, and ring-shaped enhancement on contrast-enhanced images. After treatment, the signal intensity of the lesions slightly decreased on T2WI and DWI.

**Fig. (2):**
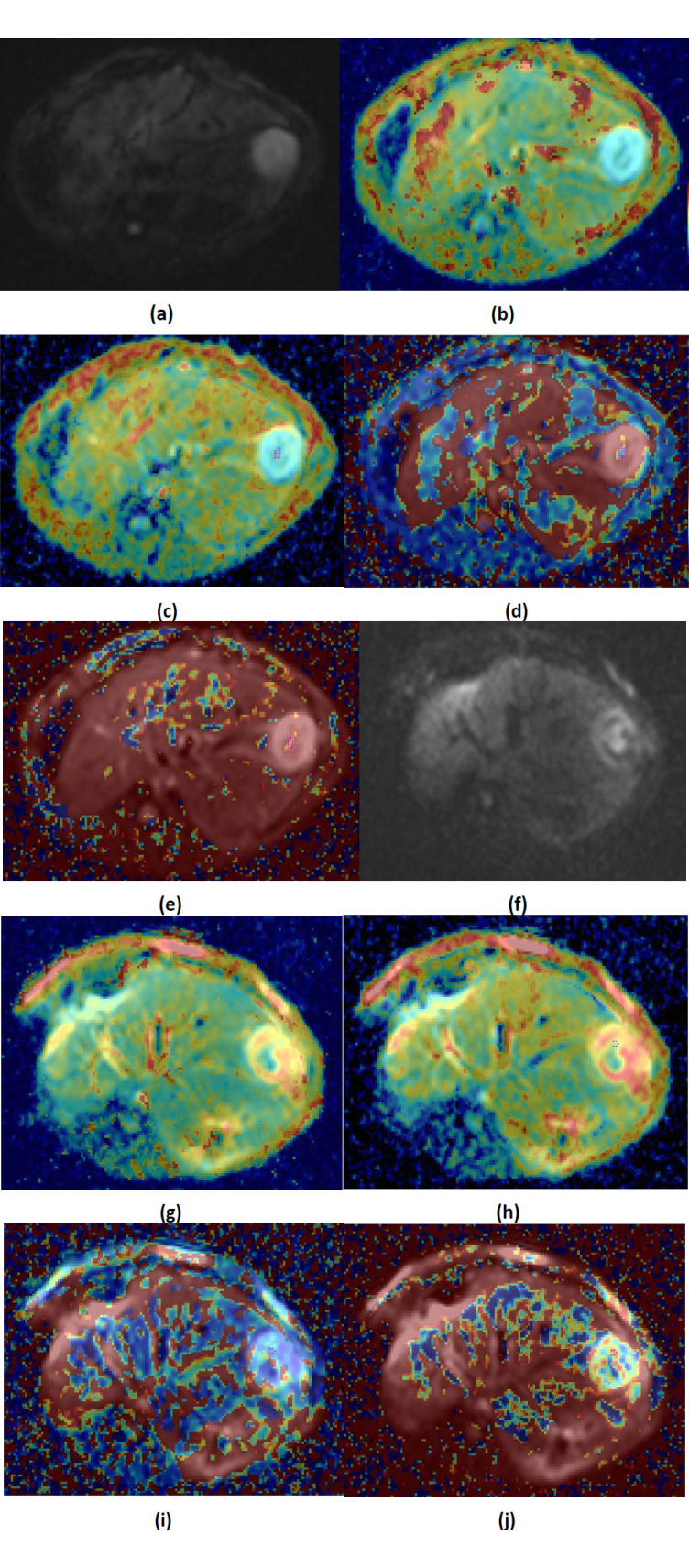
MR images of rabbit VX2 liver tumors before (**a**-**e**) and after IPTT treatment (**f**-**j**). Corresponding images are shown in DWI (**a**, **f**), ADC (**b**, **g**), D (**c**, **h**), D* (**d**, **i**), and perfusion fraction f (**e**, **j**). ADC and D values are significantly lower in viable tumor regions than in inflammatory reaction regions, whereas D* shows no significant difference between the two regions after treatment. The red marks are part of the necrotic area.

Gross specimen sections were selected to correspond to positions similar to the MRI slices, and these were compared with histopathological sections as well as the imaging findings (T1WI, T2WI, contrast-enhanced MRI, and DWI) to determine the distribution and boundaries of different tissue types within the lesion after photothermal treatment. Histopathological correlation showed that DWI identified three distinct regions within the liver tumors post-treatment: a viable tumor region with marked hyperintensity located adjacent to a hypointense necrotic region, and an inflammatory reaction region appearing as a slightly hyperintense rim surrounding the necrotic zone (Fig. **2**).

No significant change in tumor size was observed one week after treatment, as assessed by MRI. However, both the mean ADC and D values showed significant increases following treatment (*P* = 0.008 and *P* = 0.034, respectively; Table **1**).

Based on the analysis of pathology and MRI findings, three distinct regions were delineated within 30 post-treatment lesions identified on DWI: the viable tumor region, the inflammatory reaction region, and the necrotic region. The values for ADC, D, D*, and f in the viable tumor, necrotic, and inflammatory reaction regions are presented in Table **2** and illustrated in Fig. (**3**). Statistically significant differences were observed, with ADC and D values being markedly lower in the viable tumor region compared to the inflammatory reaction region (*P* < 0.001 for both). Conversely, the D* values in the viable tumor region did not exhibit significant differences when compared to the inflammatory reaction region. Furthermore, ADC, D, and f values were significantly reduced in the necrotic region relative to the inflammatory reaction region (*P* = 0.003, *P* < 0.001, and *P* < 0.001, respectively), while the D* value did not demonstrate a statistically significant difference between the inflammatory reaction and necrotic regions. Notably, only the D* parameter revealed a significant difference between the viable tumor and necrotic regions (*P* = 0.026, as shown in Table **3**).

**Fig. (4):**
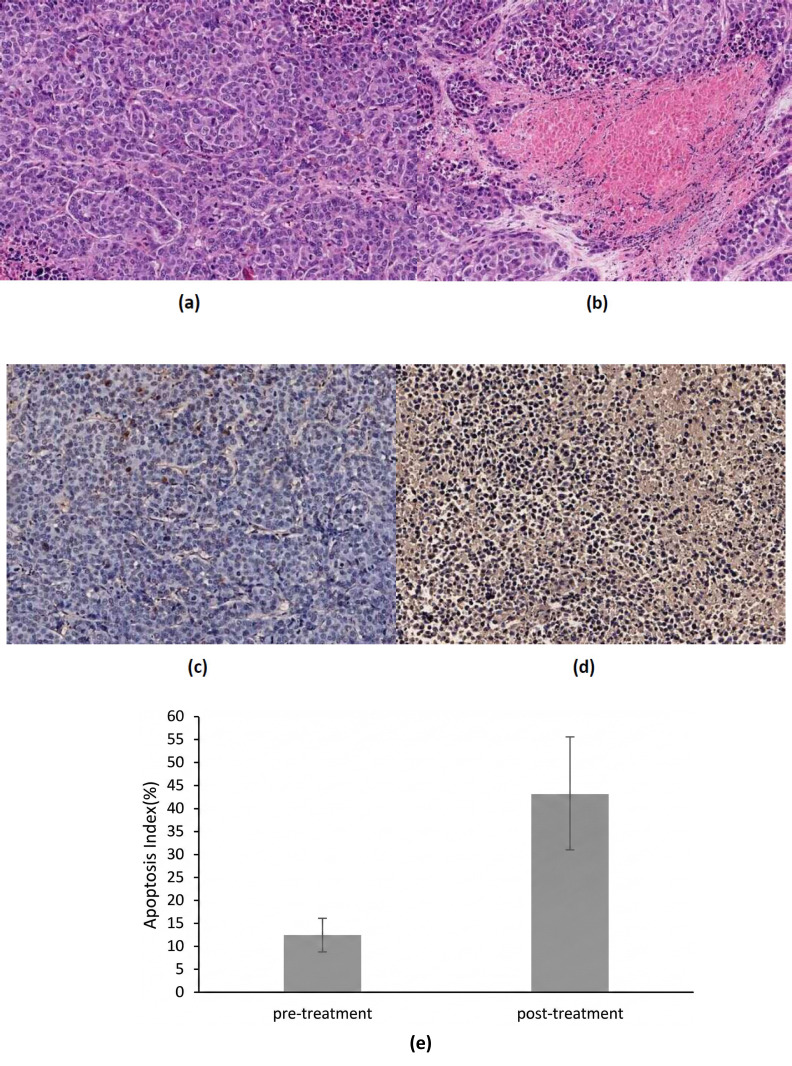
Receiver operating characteristic (ROC) curves of ADC, D, and f for differentiating between viable tumor and inflammatory reaction regions in liver tumor after treatment. The ROC curves demonstrated that D had a higher AUC than ADC and f.

ROC curves were employed to distinguish between viable tumor regions and inflammatory reaction areas in the liver following treatment. The parameter D exhibited the highest area under the ROC curve (Az = 0.867), with an optimal cutoff value of 1.16 × 10^−3^ mm^2^/s. This was followed by the ADC, which had an area under the curve (AUC) of 0.804, and D*, which demonstrated an AUC of 0.758 (Table **4** and Fig. **4**).

### 3.2 Histological and Immunohistochemical Assays

The rabbits were euthanized immediately after the MRI examination to facilitate pathological assessment. Hematoxylin–eosin–stained tumor specimens revealed a substantial area of coagulative necrosis at the center of the lesion, with some cells exhibiting nuclear condensation, karyorrhexis, and karyolysis. The inflammatory reaction area at the lesion periphery was characterized by fibroplasia or granulation tissue. HE-stained sections demonstrated a higher percentage of necrotic tissue in treated tumors compared with untreated ones.

**Fig. (5):**
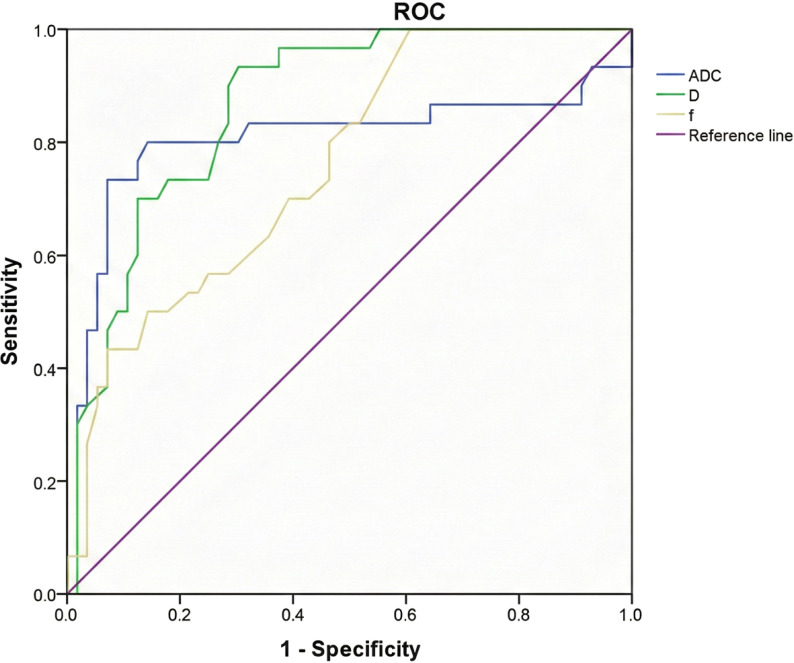
HE-stained photomicrographs of untreated (**a**) and treated (**b**) tumor; TUNEL staining of rabbit VX2 liver tumors before (**c**) and one week after IPTT therapy (**d**). The bar chart shows a statistically significant difference in the quantitative measurement of apoptotic tumor cells (**e**) (*P* <0.001).

TUNEL-stained slides were used to evaluate cell apoptosis, revealing a significant increase in the apoptosis rate from pre-treatment (12.63 ± 3.62%) to post-treatment tumors (43.26 ± 12.26%, *p* < 0.001) (Fig. **5**). All histopathological analyses were performed by an experienced pathologist.

## 4 DISCUSSION

PTT exploits the photothermal effect of photothermal transduction agents (PTAs) to eradicate various cancer types. PTAs are capable of absorbing light energy and converting it into heat, thereby elevating the local temperature and inducing cancer cell death [20-25]. Furthermore, PTAs that preferentially accumulate in tumors as opposed to surrounding healthy tissues are anticipated to enhance the efficacy of PTT [26, 27]. Typically, PTT agents are administered to tumors *via* intravenous injection. However, a significant proportion of these nanomaterials is sequestered and eliminated by the reticuloendothelial system (RES), resulting in less than 5% of the total nanomaterials ultimately accumulating in the tumors [28, 29]. Consequently, effective strategies are required to improve tumor targeting. Currently, transarterial administration (TA) is employed as a minimally invasive interventional clinical technique [30]. Using the TA technique, PTT agents can be delivered directly to tumor sites *via* the blood vessels supplying them.

Additionally, to address the limited penetration depth of NIR light, we opted to utilize real-time ultrasound-guided IPTT. As reported, the integration of IPTT, TA, and tumor mode significantly enhances tumor targeting and photothermal effects [31, 32]. In this study, we developed a tumor therapy strategy that combines the transcatheter intra-arterial infusion of Bi-based nanoparticles (Bi-Ln NPs) with IPTT of tumors. The transcatheter intra-arterial infusion technique, guided by digital subtraction angiography (DSA), was employed for transarterial administration, resulting in significantly increased accumulation in the tumors. Additionally, real-time ultrasound-guided IPTT effectively overcomes the limited penetration depth associated with NIR. The therapeutic outcomes were evaluated and analyzed using contemporary imaging techniques in a timely manner.

Initial responses to treatment are characterized by local metabolic and circulatory changes in tissue, preceding any morphological alterations. Consequently, the observation of tumor morphology changes *via* imaging techniques requires a relatively extended period. Furthermore, certain novel antitumor therapies may primarily inhibit tumor growth without significantly affecting tumor volume, highlighting limitations in evaluating therapeutic efficacy based solely on morphological changes. Conventional imaging modalities, such as MRI or CT, provide primarily morphological information, complicating the differentiation between residual tumor tissue and inflammatory responses within liver tumors post-treatment. IVIM-DWI emerges as a promising early noninvasive technique for assessing tumor metabolism following treatment. This method offers insights into tumor viability, micro-structure, and vascular distribution by detecting water proton mobility within tumor tissue [33], thereby reflecting the tissue's water content and the concentration of proteins and macromolecules. ADC and IVIM parameters have the potential to exhibit alterations before anatomical changes become apparent, thereby serving as early indicators for therapy evalu-ation and enabling differentiation between distinct regions [34].

Nevertheless, the single-index model DWI-ADC values are limited in their ability to differentiate between diffusion and perfusion information. In contrast, the double-exponential model IVIM-DWI is capable of quantitatively assessing both the true diffusion motion of water molecules and the pseudo-diffusion resulting from microcirculation perfusion by utilizing multiple b-values. This approach effectively compensates for the influence of microcirculation on DWI-ADC values [35].

Experimental investigations involving VX2 rabbits have revealed that tumor growth becomes significantly pronounced 14 days post-implantation [36]. Consequently, we initiated therapeutic intervention two weeks after implantation and performed a quantitative assessment of parameter changes both before treatment and one week after treatment. Our findings indicated that IVIM-DWI is capable of reflecting therapeutic responses in VX2 rabbits following IPTT. Notably, the ADC and D values were significantly lower in the liver tumor region compared with normal tissue, a result attributable to increased tumor cellularity, which reduces extracellular space and restricts microscopic water diffusion within tumor tissue. Previous research [37] has demonstrated that effective anticancer treatment leads to enhanced water diffusion. The results of the present study corroborate the relationship between ADC changes and post-therapy response. Furthermore, we observed increases in both mean ADC and D values following treatment.

Our study also confirmed that ADC and D values were significantly higher in regions of inflammatory reaction compared with viable tumor regions and necrotic regions. These changes may result from the loss of tumor cell membrane integrity and a subsequent decrease in cell density, allowing less restricted movement of water molecules [38]. These alterations were detectable as early as one week post-treatment, highlighting the sensitivity of ADC and D as surrogate markers for early tumor response, consistent with findings from other studies [39]. In our study, the D parameter demonstrated the best diagnostic performance, achieving the highest Az value for distinguishing viable tumor regions from areas of inflammatory reaction. The D parameter specifically reflects the motion of pure water molecules, particularly in the extracellular space [40]. In tumor tissues, ADC values were higher than D values, suggesting that the single-exponential model may overestimate water diffusion in cancerous tissues, as ADC reflects the combined effects of microscopic perfusion and diffusion. Thus, the D value provides a more accurate representation of pure water molecule movement within tissues.

The f value represents the ratio of capillary volume to total tissue volume within a voxel, and it is associated with the microvessel density (MVD) of normal or immature capillaries. This parameter can partially reflect the rate of vascular survival [41]. In the present study, f values were lower in necrotic regions compared with areas of inflammatory reaction, with a significant decrease observed after one week. This finding suggests that f may serve as a potential early indicator of post-therapy response. The decline in f values may be attributed to local fibrosis, necrosis, and angiolysis induced by high-dose thermal radiation, which disrupt microcirculation in multiple ways. This phenomenon is consistent with previous findings in the later stages of pulmonary fibrosis [42].

The D* value is associated with the microcirculatory perfusion of the capillary network in tumors, primarily reflecting the capillary flow rate within tissues. It provides insights into the perfusion status of tumors and offers quantitative data for clinical treatment [43]. In our study, D* values were found to be lower in necrotic regions compared to viable tumor regions, indicating reduced perfusion in the necrotic areas of the tumor. Theoretically, according to the double exponential model, both f and D* should exhibit similar trends. However, our findings did not demonstrate a consistent correlation between D* and f, and D* did not achieve a significant Az value for distinguishing between viable tumor and inflammatory reaction regions based on ROC analysis. This inconsistency may be attributed to the large standard deviation, data instability, and dependence on signal-to-noise ratio (SNR) associated with the D* value, which limits its broader clinical application. Similar observations have been reported in previous studies [44, 45]. Furthermore, both the values of f and D* characterize the impact of capillary microcirculation perfusion, albeit they capture distinct facets of this phenomenon. Specifically, D* is associated with the length and flow rate of the capillary network, whereas f represents the ratio of capillary volume to the total tissue volume within a voxel.

## 5 STUDY LIMITATIONS

This study has several limitations. Firstly, the sample size was relatively small, comprising only 30 rabbits in this prospective study. Nevertheless, the preliminary findings are encouraging, indicating that further research with a larger cohort is warranted. Secondly, the study used a single ROI on one axial MR image of the tumor to assess IVIM parameters. Considering the three-dimensional nature of tumors, measurements obtained from a single cross-sectional image may not fully represent the entire tumor volume. Lastly, although efforts were made to achieve precise one-to-one correspondence between pathology and imaging for each specimen, some overlap in ROI placement across different tissue regions was unavoidable.

## CONCLUSION

In conclusion, VX2 tumor-bearing rabbits were administered Bi-Ln NPs *via* transcatheter intra-arterial infusion under DSA guidance, followed by real-time ultrasound-guided IPTT and subsequent MRI evaluation to assess therapeutic efficacy. IVIM-DWI demonstrated the ability to detect early tumor responses to treatment and to differentiate between various tissue types post-treatment. The parameters derived from this imaging modality may offer preliminary insights into the physiological changes induced by therapy. The potent antitumor effects observed in this study suggest that this innovative approach is both effective and feasible for tumor therapy, thereby supporting the continued development of photothermal nanomaterials in cancer treatment applications.

## Figures and Tables

**Fig. (1):**
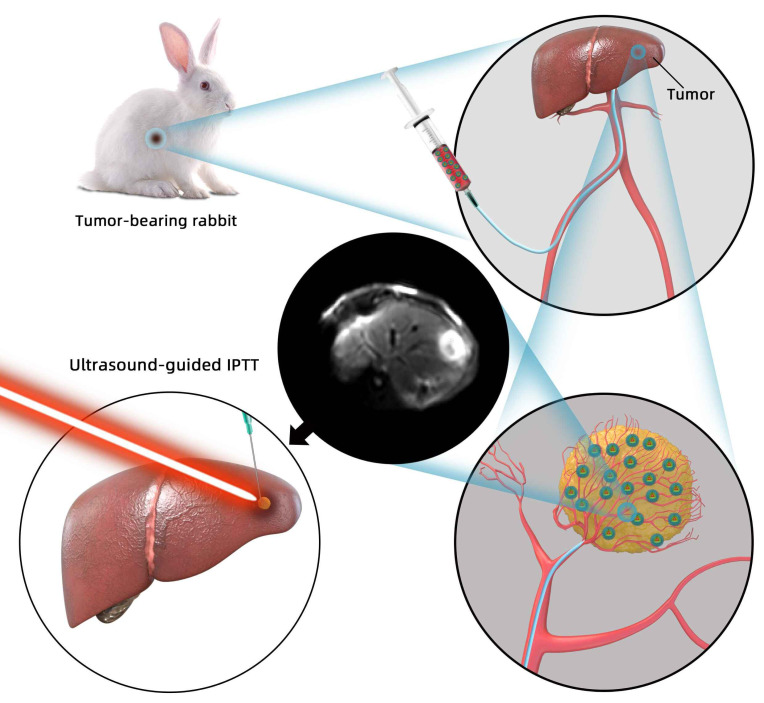
Schematic illustration of the integration of transcatheter intra-arterial infusion with IPTT for the effective eradication of hepatic tumors. Adapted from Int J Nanomedicine, 15:1373-1385, Zhou J, Ling G, Cao J, *et al*, Transcatheter Intra-Arterial Infusion Combined with Interventional Photothermal Therapy for the Treatment of Hepatocellular Carcinoma, Page No.1375, 2020 Feb 28, with permission from Zhou J, Ling G, Cao J, *et al*.

**Fig. (3):**
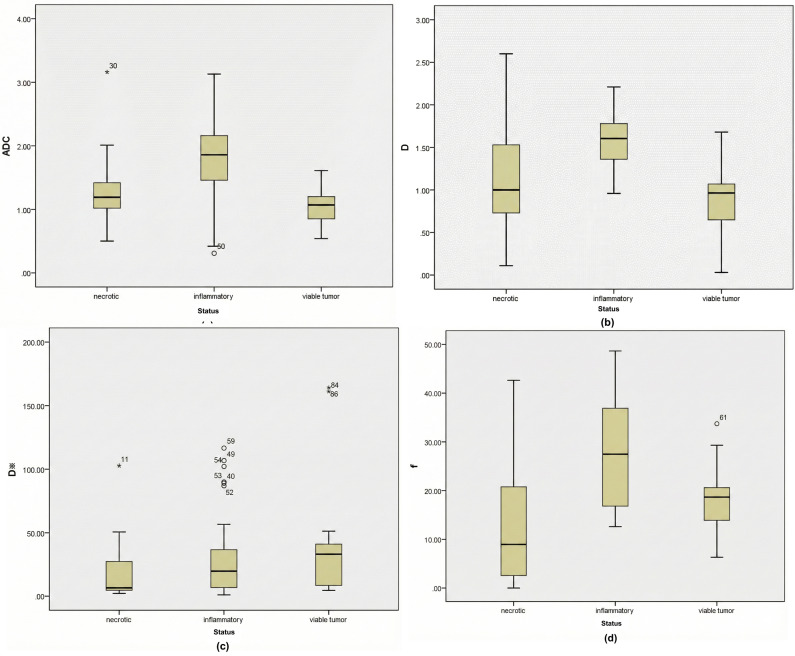
Box plots of ADC (**a**), D (**b**), D* (**c**), and perfusion fraction f (**d**) values for the necrotic, inflammatory reaction, and viable tumor regions in the liver tumor after the IPTT treatment. The midline in the box is the median value. Cross lines above and below represent the minimum and maximum values, respectively.

**Table 1:** Comparison of IVIM parameters in the field of operation before and after treatment.

**Parameter**	**ADC(×10^−3^ mm^2^/s)**	**D(×10^−3^ mm^2^/s)**	D*(×10^−3^ mm^2^/s)	**f(%)**
Prior-treatment(n=30)	1.09(0.88,1.29)	0.94(0.64,1.04)	28.68(6.76,40.21)	9.23(2.50,31.40)
Post-treatment(n=30)	1.23(1.07,1.61)	1.03(0.92,1.38)	16.78(4.7,36.26)	16.83(13.35,20.61)
*P*-value	0.008*	0.034*	0.147	0.225

**Note:** * Results indicated a significant difference (*P*<0.05).

**Table 2:** Mean values of measured parameters for three regions of post-treatment liver tumor.

**Parameter**	**Inflammatory(n=30)**	**Necrotic(n=30)**	**Viable(n=26)**
ADC(×10^−3^ mm^2^/s)	1.86(1.46,2.17)	1.19(1.02,1.43)	1.07(0.84,1.22)
D(×10^−3^ mm^2^/s)	1.61(1.36,1.79)	1.00(0.72,1.53)	0.97(0.65,1.08)
D*(×10^−3^ mm^2^/s)	19.80(6.76,41.64)	6.57(4.62,27.98)	33.12(8.08,41.36)
f(%)	27.45(16.80,37.15)	8.95(2.50,22.27)	18.65(13.85,20.61)

**Note:** Data are expressed as median (upper and lower quartiles).

**Table 3:** Results of multiple comparisons among the three regions of post-treatment liver tumor.

**Parameter**	**Inflammatory-Viable**	**Necrotic-Inflammatory**	**Necrotic-Viable**
ADC	<0.001*	0.003*	0.371
D	<0.001*	<0.001*	0.574
D*	1.000	0.175	0.026*
f	0.009*	<0.001*	0.156

**Note:** Data are *P*-values. * Results indicated a significant difference (*P*<0.05).

**Table 4:** ROC analysis differentiating between viable tumor and inflammatory reaction regions after treatment.

**Parameter**	**AUC**	**Best Cutoff**	**Sensitivity**	**Specificity**	***P*-value**
ADC(×10^−3^ mm^2^/s)	0.804(0.682,0.926)	1.435	0.8	0.857	<0.001
D(×10^−3^ mm^2^/s)	0.867(0.792,0.942)	1.16	0.933	0.696	<0.001
f (%)	0.758(0.656,0.860)	12.575	1	0.393	<0.001

**Note:** ROC, receiver operating characteristic; AUC, area under the curve; * Values in parentheses are 95% confidence intervals.

## Data Availability

The data and supportive information are available within the article.

## LIST OF ABBREVIATIONS

DSA: Digital Subtraction Angiography
NIR: Near-Infrared
MRI: Magnetic Resonance Imaging
T1WI: T1-Weighted Imaging
T2WI: T2-Weighted Imaging
ADC: Apparent Diffusion Coefficient
D: Diffusion Coefficient
ROC: Receiver Operating Characteristic
